# Supplementary material for: Oxidation of Cyclohexane/Cyclohexanone Mixture with Oxygen as Alternative Method of Adipic Acid Synthesis
Source: Materials (Basel). 2022 Dec 28;16(1):298. doi: 10.3390/ma16010298 (PMC9821978; doi:10.3390/ma16010298)
Supplement: Supplementary file 1 [file materials-16-00298-s001.zip › materials-1999655-supplementary.pdf]

materials MDPI

*Supplementary Materials*

# Oxidation of Cyclohexane/Cyclohexanone Mixture with Oxygen as Alternative Method of Adipic Acid Synthesis

**Dawid Lisicki [1,*], Beata Orlińska [1], Adam A. Marek [1], Jakub Bińczak [1,2], Krzysztof Dziuba [2] and Tomasz Martyniuk [2]**

[1] Department of Chemical Organic Technology and Petrochemistry, PhD School, Silesian University of Technology, Akademicka 2A, 44-100 Gliwice, Poland
[2] Grupa Azoty Zakłady Azotowe, "Puławy" S.A., Al. Tysiąclecia Państwa Polskiego 13, 24-110 Puławy, Poland
* Correspondence: dawid.lisicki@polsl.pl

## 1. Description of the reactor

Pressure reactor had a 100 mL capacity and was purchased from Autoclave Engineers Inc. USA. made of Hastelloy C-276 steel. The maximum working pressure was 20 MPa and the temperature set at 315 °C. The reactor was equipped with a mechanical agitator with a speed of up to 2000 rpm, electric heating jacket, pressure regulating valve, digital temperature, pressure and mixing regulator, an intermediate pressure cylinder maintaining constant pressure in the reactor.

## 2. The oxidation process description

Appropriate amounts of cyclohexane, cyclohexanone and, optionally, a solvent were introduced into the reactor, where the total amount was 20 mL, as well as the catalysts. Thereafter, the reactor was purged with oxygen/air twice, in which oxygen/air was introduced into the reactor until a pressure of 0.4 MPa was reached, followed by slow depressurization. The contents of the reactor were stirred at 200 rpm and heated at a predetermined reaction temperature (70–100 °C) at a rate of about 5 °C / min. Then, the oxidizing agent was introduced into the reactor under a specified pressure (1–2 MPa) and the mixing speed was increased to 1000 rpm.

The oxidation reaction was carried out without the flow of the oxidizing agent for a specified time (2–8 h) from the moment of reaching the set temperature. When the pressure decreased during the reaction it was replenished automatically via the introduction of $O_2$. During the reaction, the temperature, pressure in the reactor, stirring rate, and pressure drop in the intermediate cylinder were monitored, which enabled the observation of $O_2$ consumption.

After the reaction, the reactor contents were cooled to <5 °C and depressurized. Due to precipitation of crude adipic acid, 40 mL of acetic acid was charged to the reactor allowing transfer of the reaction mixture to the receiver. In most cases, the reaction products at ambient temperature were a two-phase (liquid/solid) system.

In order to perform analysis, it was necessary to homogenize the post-reaction mixture. The reaction products were heated in a two-necked flask at a volume of 100 mL, with vigorous stirring at 1000 rpm, under reflux conditions until complete dissolution of the precipitate was achieved. In most cases, a temperature of 60 °C was sufficient to obtain a homogeneous mixture, which was sampled hot for further analysis. In the reaction products, the amount of unreacted cyclohexane and cyclohexanone as well as the obtained cyclohexanol, adipic, glutaric and succinic acid were determined by GC analysis.

## 3. Procedures for the determination of individual substances using GC-FID

The composition of the post-reaction mixture was determined by the internal standard method (toluene-standard) using an Agilent Technologies 7890C gas chro-

matograph, equipped with an FID detector, autosampler, ZB-5HT column (30 m × 0.25 mm × 0.25 μm) and helium as the carrier gas.

*3.1. Determination of cyclohexane*

4 mL of the sample and 4 mL of the standard solution (both weighed with an accuracy of 0.001 g) were mixed and the composition of the solution was examined via GC. Based on the previously prepared standard curve, the amount of cyclohexane in the reaction products was determined.

Preparation of the standard solution: To a 250 mL volumetric flask, 5.63 g of toluene (weighed to the nearest 0.001 g) was introduced. The flask was made up to with acetic acid and mixed thoroughly.

Dispenser temperature: 200 °C, detector temperature: 250 °C, split: 200:1, injection: 1 μL, air: 400 ml/min, nitrogen: 24 ml/min, hydrogen: 30 ml/min, oven temperature program: 50 °C for 8 min, 10 °C/min 50–80 °C, 20 °C/min 80–220 °C, 220 °C for 4 min

*3.2. Determination of cyclohexanol and cyclohexanone*

1 ml of the sample and 5 mL of the standard solution (both weighed with an accuracy of 0.001 g) were mixed and the composition of the solution was examined by GC. On the basis of the previously prepared standard curve, the amount of cyclohexanol in the reaction products was determined.

Preparation of the standard solution: To a 250 mL volumetric flask, 5.63 g of toluene (weighed to the nearest 0.001 g) was introduced. The flask was made up to the mark with acetic acid and mixed thoroughly.

Dispenser temperature: 200 °C, detector temperature: 300 °C, split: 100:1, injection: 1 μL, air: 400 ml/min, nitrogen: 24 ml/min, hydrogen: 30 ml/min, oven temperature program: 70 °C for 10 min, 6 °C/min 70–112 °C, 20 °C/min 112–212 °C, 212 °C for 8 min.

*3.3. Determination of adipic, glutaric and succinic acids in ester form*

In order to determine the amount of obtained carboxylic acids by GC, it was necessary to carry out the esterification reaction with methanol to give dimethyl esters.

Esterification: In a 25 mL round bottomed flask, 1 mL of the sample (weighed to the nearest 0.001 g), 12 mL of toluene solution in methanol and 3–5 drops of sulfuric acid(VI) were added. The esterification reaction was stirred at 300 rpm at ambient temperature for 24 h (yield >98.5%). The composition of the solution was examined using GC - method C. On the basis of the previously prepared standard curve, the amount of adipic, glutaric and succinic acid in the reaction products was determined.

Preparation of a solution of toluene in methanol: 14.71 g of toluene (weighed with an accuracy of 0.001 g) was added to a 1 L volumetric flask. The flask was made up to the mark with methanol and mixed thoroughly.

Dispenser temperature: 200 °C, detector temperature: 300 °C, split: 100:1, injection: 1 μL, air: 400 ml/min, nitrogen: 24 ml/min, hydrogen: 30 ml/min, oven temperature program: 70 °C for 10 min, 6 °C/min 70–112 °C, 20 °C/min 112–212 °C, 212 °C for 8 min. The error of the chromatographic analysis was found to be 2%.
